# Anthracycline and antimetabolite, trigger distinct resistance mechanisms in *TP53*^mut^ AML

**DOI:** 10.1038/s41420-026-03352-z

**Published:** 2026-09-17

**Authors:** Sanjeev Raghuwanshi, Andrei L. Gartel

**Affiliations:** University of Illinois at Chicago, Department of Medicine, Chicago, IL USA

**Keywords:** Acute myeloid leukaemia, Acute myeloid leukaemia

**To the Editor**,

Acute myeloid leukemia (AML) is a highly heterogeneous and extremely aggressive form of blood cancer. Despite recent advances, AML continues to be a challenging disease to treat; the overall prognosis and response to therapy are strongly influenced by karyotypic and molecular alterations [1]. Mutations of the *Tp53* gene (*TP53*^mut^) are among the most common alterations found in human malignancies, affecting 5–20% of de novo AML and more frequently (up to 37%) observed in patients with therapy-related AML [1, 2]. The *TP53*^mut^ AML has also been associated with a lower response to conventional chemotherapies, high relapse, and inferior overall survival [2–4]. Since 2017, the Food and Drug Administration (FDA) has approved several new drugs for AML [1], but treating *TP53*^muts^ AML remains a formidable challenge.

The conventional cytotoxic therapy based on DNA-damaging agents, particularly anthracyclines, including idarubicin (IDR) and daunorubicin (DNR), combined with cytarabine (Ara-C), remains one of the cornerstones in AML management [1, 3]. But these regimens result in dismal clinical outcomes in *TP53*^mut^ AML, possibly due to the emergence of resistance [2–4]. Currently, the mechanisms by which *TP53*^muts^ mediate resistance to DNA-damaging drugs are poorly understood. Therefore, the focus is on identifying molecular targets and the most effective combination of existing chemotherapies with targeted therapies for greater efficacy in patients with *TP53*^mut^ AML.

The activation of the DNA damage response (DDR) is one of the main mechanisms by which cancer cells cope with high levels of oncogene-driven replication stress and becomes essential for survival after DNA-damaging drug induction [5, 6]. On the other hand, anti-apoptotic BCL2 family proteins promote AML survival and therapy resistance by sequestering pro-apoptotic proteins [7]. To aid clinical studies aiming to design effective combination therapies for patients with *TP53*^mut^ AML, we conducted a systematic study to understand resistance mechanisms induced in response to different DNA-damaging drugs.

FOXM1, a member of the Forkhead family of transcription factors, is aberrantly high-expressed in AML and promotes cancer survival by regulating multiple functions, such as facilitating DNA damage repair and preventing apoptosis [8]. In AML with NPM1 mutations [1], the transcriptional inactivation of FOXM1 in the cytoplasm confers favorable treatment outcomes for AML patients [9]. In the present study, pharmacological inhibition of FOXM1 by STL001, a selective FOXM1 inhibitor [9], did indeed sensitize *TP53-*altered AML cells (*TP53*^mut^ KG-1 and *TP53-*null HL-60) to the cytotoxic effect of different DNA-damaging drugs (IDR, DNR, Ara-C, and 6-TG), as evidenced by increased caspase-3 cleavage (Fig. 1A, B). Results indicate that FOXM1 drives resistance to DNA-damaging drugs in *TP53*^mut/null^ AML cells, though the underlying mechanisms remain to be elucidated.

**Fig. 1:**
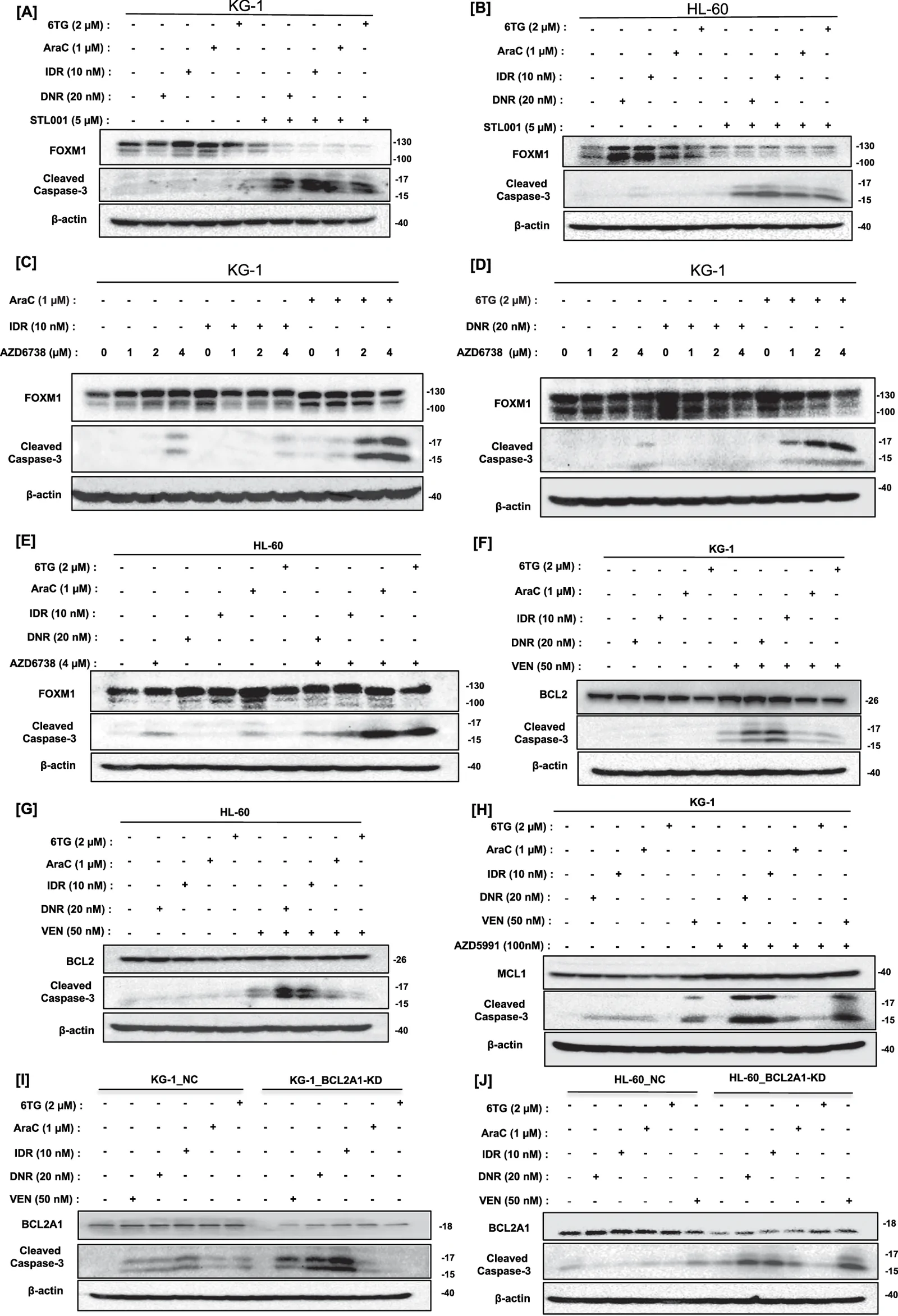
In TP53^mut^ AML, standard chemotherapies based on anthracyclines (IDR and DNR) and antimetabolites (Ara-C and 6-TG) face distinct resistance mechanisms. **A**, **B** KG-1 and HL-60 cells were treated with indicated concentrations of DNR, IDR, Ara-C, or 6-TG alone and in combination with STL001 for 24 h. In all cases, total protein samples were obtained from cells immediately after treatment and analyzed for FOXM1 and cleaved caspase-3 levels *via* immunoblotting, and β-actin was used as an internal loading control. **C**, **D** KG-1 cells were treated with increasing concentrations of the ATR inhibitor AZD6738 alone and in combination with indicated concentrations of DNR, IDR, Ara-C, or 6-TG for 24 h; [**E**] HL-60 cells were treated with indicated concentrations of DNR, IDR, Ara-C, and 6-TG alone or in combination with AZD6738 for 24 h. In all cases, total protein samples were obtained from cells immediately after treatment and analyzed for FOXM1 and cleaved caspase-3 levels *via* immunoblotting, and β-actin was used as an internal loading control. **F**, **G** KG-1 and HL-60 cells were treated with  the indicated concentrations of DNR, IDR, Ara-C, or 6-TG alone and in combination with venetoclax (VEN) for 24 h. In all cases, total protein samples were obtained from cells immediately after treatment and analyzed for BCL-2 and cleaved caspase-3 levels *via* immunoblotting, and β-actin was used as an internal loading control. **H** KG-1 cells were treated with indicated concentrations of DNR, IDR, Ara-C, or 6-TG alone and in combination with MCL-1 inhibitor (AZD5991) for 24 h. In all cases, total protein samples were obtained from cells immediately after treatment and analyzed for MCL-1 and cleaved caspase-3 levels *via* immunoblotting, and β-actin was used as an internal loading control. **I**, **J** KG-1 and HL-60 cells with stable shRNA–mediated BCL2A1-KD or a control vector were treated with the indicated concentrations of DNR, IDR, Ara-C, 6-TG, or venetoclax (VEN). In all cases, total protein samples were obtained from cells immediately after treatment and analyzed for BCL2A1 and cleaved caspase-3 levels via immunoblotting, and β-actin was used as an internal loading control. The blots shown are representative of three independent experiments with consistent results.

Since anthracycline and the antimetabolite group of cytotoxic chemotherapies exert cytotoxic effects mainly through DNA damage [5], resistance may be governed by the FOXM1-AKT loop through regulating the DDR (ATM/ATR) pathways [10, 11]. Therefore, we first assessed antileukemic interactions between ATM or ATR inhibitors and different DNA-damaging drugs in *TP53*^mut/null^ AML cells. We treated KG-1 and HL-60 cells with increasing concentrations of the ATM inhibitor (M3541) and ATR inhibitor (AZD6738), alone or in combination with sublethal concentrations of different DNA-damaging drugs, including IDR, DNR, Ara-C, and 6-TG. After 24 hours of drug treatment, cells were subjected to immunoblot analysis. As shown in Fig. 1C–E, the ATR inhibitor alone generated no adverse effect on FOXM1 expression levels and did not exert prominent cytotoxic action on its own. Furthermore, it did not sensitize AML cells to anthracyclines (IDR and DNR). However, when combined with antimetabolites (Ara-C or 6-TG), it led to a potent induction of apoptosis, as indicated by caspase-3 cleavage (Fig. 1C–E). This provides a rationale for combining ATR inhibitors with antimetabolites to overcome resistance in high-risk *TP53*^mut/null^ AML. In contrast, the ATM inhibitor did not sensitize *TP53*^mut/null^ AML cells to anthracyclines or antimetabolites (Supplementary Fig. 1). Anthracyclines induce DNA DSBs by poisoning topoisomerase II [12]; DNA DSBs activate primarily the ATM/p53 response to induce cell-cycle arrest, which enables cells to repair damaged DNA [12, 13]. Unlike anthracyclines, antimetabolites induce stalled replication forks, triggering the ATR-CHK1 DNA replication stress response pathway, which stabilizes replication forks and allows cell-cycle progression and cell survival in a p53-independent manner [13]. In our study, ATR inhibition sensitized AML cells to apoptosis induced by antimetabolites (Fig. 1C–E), most likely by causing the accumulation of stalled forks, which in turn favors fork collapse and the induction of p53-independent apoptosis [13]. However, ATR inhibition failed to sensitize AML cells to anthracyclines, suggesting that the ATR-CHK1 pathway is not primarily involved in the response to anthracycline-induced DNA DSBs [12, 13]. Similarly, ATM inhibition did not sensitize AML cells to antimetabolites, most likely because the ATM/p53 pathway is not the primary mechanism managing antimetabolite-induced replication stress [12, 13]. Since the ATM inhibitor also failed to sensitize *TP53*^mut/null^ AML cells to anthracycline-induced apoptosis [12], we postulate an alternative survival mechanism in response to DNA damage caused by anthracyclines [6].

Pro-survival members of the BCL2 family are often associated with resistance to chemotherapies in AML [7, 8]. The dose-dependent increase in BCL2 expression following anthracycline treatment (Supplementary Fig. 2) suggests that anti-apoptotic BCL2 family proteins could be associated with increased tolerance to DNA damage induced by anthracyclines. Venetoclax is a potent and selective small-molecule BCL2 inhibitor that has been studied in several hematologic malignancies both as monotherapy and in combination with other agents [1]. While venetoclax plus low-dose Ara-C (VEN/LDAC) has demonstrated efficacy in de novo AML and received U.S. FDA approval for the treatment of older/ineligible AML patients based on the VIALE-C study (NCT03069352) [14], this regimen has not meaningfully overcome the adverse-risk biology of *TP53*^mut^ AML [4, 15]. These studies highlight the need to understand the interactions of different DNA-damaging drugs with inhibitors of pro-survival BCL2 family proteins. Thus, we sought to investigate whether the BCL2 inhibitor venetoclax sensitizes *TP53*^mut/null^ AML cells to different DNA-damaging drugs. We treated KG-1 and HL-60 cells with sublethal concentrations of anthracyclines (IDR and DNR) and antimetabolites (Ara-C and 6-TG) alone or in combination with the BCL2-inhibitor venetoclax. As shown in Fig. 1F, G, venetoclax treatment did not affect BCL2 expression levels but prominently enhanced the cytotoxic activity of anthracyclines and sensitized AML cells to anthracycline-induced apoptosis, as indicated by caspase-3 cleavage. In contrast, the addition of venetoclax to antimetabolite drug treatments did not show any sensitization effect in AML cells (Fig. 1F, G).

Further, we have investigated the dependencies of AML cells on alternative anti-apoptotic BCL-2 family proteins (MCL-1 and BCL2A1) in response to DNA damage induction [7]. We treated KG-1 and HL-60 cells with sublethal concentrations of different DNA-damaging drugs alone or in combination with the MCL-1 inhibitor AZD5991. While AZD5991 increases overall MCL-1 (Fig. 1H), its effect is attributed to the direct inhibition of MCL-1 activity. In this study, the MCL-1 inhibitor sensitized AML cells only to anthracycline-induced apoptosis, as indicated by caspase-3 cleavage in immunoblot analysis; however, it did not show prominent cytotoxic action when combined with antimetabolites (Fig. 1H).

Recently, we and others have shown that BCL2A1, a member of the anti-apoptotic BCL2 family proteins, regulates cell survival by conferring resistance to BCL2 family inhibitors [16]. Thus, we sought to assess whether BCL2A1 confers resistance to different DNA-damaging drugs used in this study. In the absence of a pharmacologic inhibitor of BCL2A1, we treated BCL2A1-KD AML cells (KG-1 and HL-60) with sublethal concentrations of anthracyclines, antimetabolites, or venetoclax. Consistent with our findings using BCL2 and MCL-1 inhibitors, BCL2A1-KD sensitized AML cells to anthracycline-induced apoptosis, indicated by caspase-3 cleavage (Fig. 1I, J). Notably, BCL2A1-KD did not sensitize cells to antimetabolite-induced apoptosis (Fig. 1I, J). These results suggest that targeting alternative BCL-2 family members (BCL2, BCL2A1, and MCL-1) can effectively sensitize AML cells to anthracyclines.

Furthermore, we have validated these sensitization results by systematically measuring the interactions between DNA-damaging drugs and selective BCL2 or MCL-1 inhibitors. In these studies, we used a dose-response matrix with TP53^mut^ AML cells (KG-1) treated with venetoclax or AZD5991 and different DNA-damaging drugs (Fig. 2). In our study, both the BCL2 and MCL-1 inhibitors exhibited strong synergistic interactions with anthracyclines (IDR and DNR) in reducing the viability of AML cells (Fig. 2A, B, E, F). As shown in Fig. 2, ZIP (zero interaction potency) synergy scores of approximately 20 or above indicated robust synergy in growth suppression and demonstrated that inhibition of BCL2 or MCL1 could effectively sensitize cells to the growth-obstructing actions of anthracyclines (IDR or DNR). In contrast, combining BCL2 or MCL-1 inhibitors with antimetabolites (Ara-C and 6-TG) did not exhibit pronounced synergistic interactions in reducing the viability of AML cells (Fig. 2C, D, G, H). As shown in Fig. 2, ZIP synergy scores between 2 and9 generally indicate that these drugs have additive or no interactions in growth suppression when combined with BCL2 or MCL-1 inhibitors. The observed sensitization to anthracyclines by blockade of alternative BCL2 family members (BCL2, BCL2A1, and MCL-1) appears to be driven by the strong synergy between anthracyclines and BCL2 family inhibitors.

**Fig. 2:**
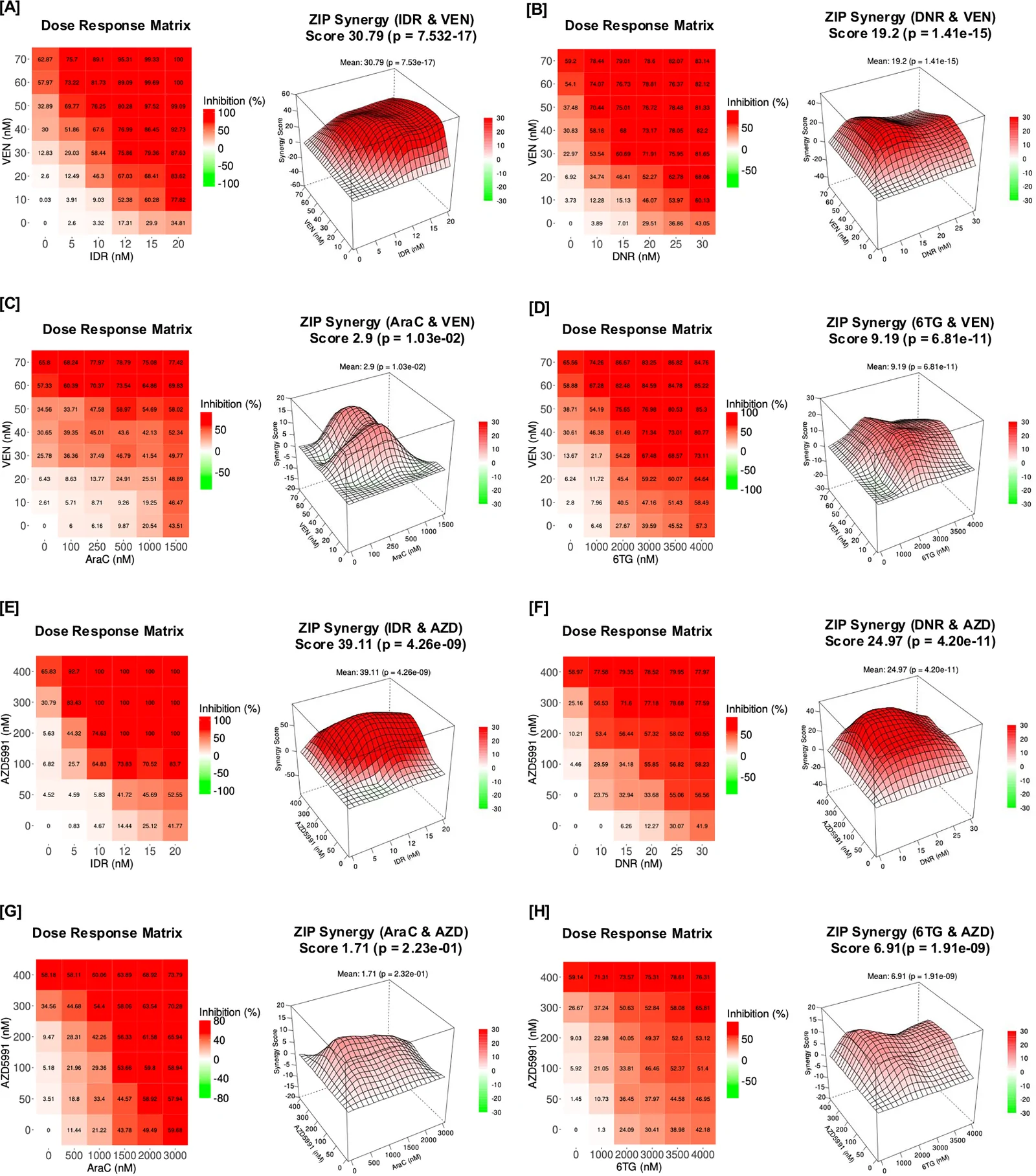
Assessment of the interactions in growth suppression between inhibitors of BCL2 family proteins and different DNA-damaging drugs. Dose-response matrix and ZIP synergy score surface plots of KG-1 cells treated with (**A**) Venetoclax (VEN) plus Idarubicin (IDR), (**B**) VEN plus Daunorubicin (DNR), (**C**) VEN plus cytarabine (Ara-C) or (**D**) VEN plus 6-thioguanine (6-TG), and (**E**) AZD5991 plus IDR, (**F**) AZD5991 plus DNR, (**G**) AZD5991 plus Ara-C, or (**H**) AZD5991 plus 6-TG. Cells were treated for 24 h with each drug alone or in combination at each dose pair. An inhibition matrix and ZIP synergy matrix are shown for the combination of drugs. The ZIP synergy score is the average synergy score of all dose pairs in the matrix, with a 95% confidence interval, 3 replicates per treatment.

In summary, these data indicate that DNA-damaging drugs, anthracyclines (IDR and DNR), and antimetabolites (Ara-C and 6-TG) face distinct chemoresistance in *TP53*^mut^ AML. Our data provide evidence that inhibitors of anti-apoptotic BCL2 family proteins demonstrate no synergy with antimetabolites (Ara-C and 6-TG), potentially explaining why VEN/LDAC failed to improve outcomes in patients with *TP53*^mut^ AML [4, 15]. Our findings suggest that we need novel therapies beyond BCL2 inhibitors to overcome resistance to antimetabolites, and support the further development of ATR inhibitors in combination with antimetabolites for *TP53*^mut^ AML. In contrast, inhibitors of the anti-apoptotic BCL2 family members (BCL2 and MCL1) demonstrate strong synergistic interactions with anthracyclines (IDR and DNR), suggesting that the pro-survival BCL2 family is the major driver of resistance to anthracycline-based chemotherapies. While our study is limited by its in vitro design, these data serve as a hypothesis-generating foundation to guide future functional studies, ultimately aiding in finding the right combination of drugs and novel therapeutic approaches for *TP53*^mut^ AML.

## Supplementary information

suppl original blots

Suppl

## Data Availability

The data supporting the findings of this study are available within the article.
