# Supplementary material for: Anthracycline and antimetabolite, trigger distinct resistance mechanisms in TP53mut AML
Source: Cell Death Discov. 2026 Sep 17;12:375. doi: 10.1038/s41420-026-03352-z (PMC13586370; doi:10.1038/s41420-026-03352-z)
Supplement: Supplementary file 1 [file 41420_2026_3352_MOESM1_ESM.docx]

**Original Data.** Full uncropped images of blots shown in the paper (Figure 1A-J). For each immunoblot image in the paper, molecular weights of protein markers are indicated on the right.

**Figure1A**

**Figure1B**

**Figure 1C**

**Figure 1D**

**Figure 1E**

**Figure 1F**

**Figure 1G**

**Figure 1H**

**Figure 1I**

**Figure 1J**
