# Supplementary material for: Anthracycline and antimetabolite, trigger distinct resistance mechanisms in TP53mut AML
Source: Cell Death Discov. 2026 Sep 17;12:375. doi: 10.1038/s41420-026-03352-z (PMC13586370; doi:10.1038/s41420-026-03352-z)
Supplement: Supplementary file 2 [file 41420_2026_3352_MOESM2_ESM.docx]

**Supplementary Information**

**Anthracycline and Antimetabolite, Trigger Distinct Resistance Mechanisms in *TP53*^mut^ AML**

**Sanjeev Raghuwanshi^1^, Andrei L Gartel^1^***

**1 University of Illinois at Chicago, Department of Medicine, Chicago, IL, USA**

***Corresponding authors: Andrei L Gartel1* Email: agartel@uic.edu**

**Inventory of Supplemental Information**

**Supplemental Methods**

**Supplemental Figures and Table**

**Methods:**

**Cell Cultures**

The present study was conducted with the *TP53*^mut/null^ human acute myeloid leukemia (AML) cells (KG-1 and HL-60) obtained from the American Type Culture Collection (Manassas, VA, USA). Cells were cultured in IMDM with 2.0 mM L-glutamine (Thermo Fisher Scientific, Waltham, MA, USA) supplemented with 10% fetal bovine serum, 50 U/mL Penicillin, and 50 µg/mL Streptomycin (Gibco, Thermo Fisher Scientific). AML cells were maintained in a humidified CO2 incubator at 37°C with 5% CO2 and confirmed to be mycoplasma-negative by PCR-based routine testing.

**Chemical Compounds and Drugs**

STL001 (Vitas-M Laboratory, Hong Kong), Daunorubicin hydrochloride, Idarubicin hydrochloride, 6-Thioguanine (LKT Laboratories, USA), M3541, AZD6738, AZD5991, venetoclax (Selleck Chemicals, Houston, TX, USA), and Cytarabine (Millipore Sigma) were dissolved in dimethyl sulfoxide (MilliporeSigma). Geneticin (Gold Biotechnologies) was dissolved in sterile water.

**Stable BCL2A1-expression KD**

AML cells (HL-60 and KG-1) at 75% confluency were seeded into 12-well tissue culture plates and transduced with the MISSION pLKO.1-NEO lentiviral particles expressing nontargeting control or BCL2A1 shRNA (Millipore Sigma) in the presence of Polybrene (10 μg/ml) at a multiplicity of infection (MOI) of 10. Following overnight incubation in a humidified incubator at 37 °C, transduced cells were selected by cultivation in presence of geneticin (at 500 μg/ml). An untransduced control plate was used to determine when the selection was complete.

**Drug Treatment of Cultured Cells**

AML Cells (KG-1 and HL-60) were grown at 37°C in a humidified CO2 incubator. Following centrifugation at 200 g for 5 minutes, viable cells were counted using the Trypan Blue (0.4%) dye exclusion test (Thermo Fisher Scientific). Cultures with >90% viability were used for all treatments. In different experimental conditions, AML cells were resuspended in IMDM growth medium (1.5 x 10^6^ viable cells/ well of a 6-well tissue culture plate) with selected concentrations of drugs. Control groups were treated with vehicle only (DMSO concentration < 0.3%). Following 24 hours of incubation, all samples were collected by centrifugation and washed with ice-cold PBS and processed for immunoblotting as described below. A sample size of (n=3) biological replicates was used, and treatments were randomly assigned to wells in all experiments.

**Protein Immunoblotting**

The cells were collected and washed with ice-cold PBS by centrifugation; total protein was extracted with brief sonication in ice-cold RIPA lysis buffer (Thermo Fisher Scientific) and protease inhibitor cocktail (Thermo Fisher Scientific). The proteins were separated by 4-12% SDS-polyacrylamide gels and transferred to Immobilon-Psq PVDF membranes (Millipore Sigma). Membranes were blocked using 5% milk in Tris-buffered saline solution (TBS) with 0.05% Tween-20 (TBST) for 1 hour at room temperature. After washing with TBST, membranes were probed with specific primary antibodies (Supplementary table 1) overnight at 4°C. Membranes were washed with TBST and further incubated with Horseradish Peroxidase (HRP) conjugated secondary antibodies (Supplementary table 1) for one hour at room temperature. After washing with TBST, protein bands on membranes were developed using SuperSignal West Pico ECL Substrate (Thermo Fisher Scientific) on the ChemiDoc MP System (Bio-Rad).

**Cell viability and synergy analyses**

The cell proliferation assay WST (Abcam) was used to quantify cell viability [1]. The cells were treated with different concentrations of BCL2/MCL1 inhibitors and DNA damaging drugs (DNR, IDR, Ara-C, and 6-TG) alone or in combination for 24 hours. Assays were performed in triplicate following instruction manual and the data statistics was analyzed by the PRISM. Synergy between BCL2/MCL1 inhibitors and different DNA damaging drugs (DNR, IDR, Ara-C, and 6-TG) in suppression of cell viability was assessed using Zero Interaction Potency (ZIP) synergy score model, with the help of Synergy plus online tool (<https://www.synergyfinderplus.org/>) [2]. The ZIP model uses bootstrapping for statistical analysis.

**References:**

1. **Kumar S, Tchounwou PB. p53 as a unique target of action of cisplatin in acute leukaemia cells. J Cell Mol Med. 2022 Sep;26(17):4727-4739.**
2. **Zheng, S.; Wang, W.; Aldahdooh, J.; Malyutina, A.; Shadbahr, T.; Tanoli Z; Pessia, A.; Tang, J. SynergyFinder Plus: Toward Better Interpretation and Annotation of Drug Combination Screening Datasets. Genomics, Proteomics & Bioinformatics 2022, 20, 587-596**

**Supplementary Figure 1**

**Supplementary Figure 1. ATM inhibitor (M3541) does not sensitize Tp53^mut^ AML cells to Idarubicin (IDR) or cytarabine (Ara-C).** KG-1 cells were treated with indicated concentrations of IDR and Ara-C alone and in combination with M3541 for 24 h. In all cases, total protein samples were obtained from cells immediately after treatment and analyzed for FOXM1 and cleaved caspase-3 levels *via* immunoblotting, and β-actin was used as an internal loading control. The blot shown is representative of three independent experiments with consistent results.

**Supplementary Figure 2**

**Supplementary Figure 2. Idarubicin (IDR) but not cytarabine (Ara-C) increases BCL2 expression levels in concentration dependent manner.** KG-1 cells were treated with increasing concentrations of, IDR and Ara-C for 24 h. In all cases, total protein samples were obtained from cells immediately after treatment and analyzed for BCL2 and cleaved caspase-3 levels *via* immunoblotting, and β-actin was used as an internal loading control. The blot shown is representative of three independent experiments with consistent results.

**Supplementary table 1. List of antibodies used in immunoblotting.**

| **S.No.** | **Protein target** | **Supplier and cat. #** | **clone/ID and host** | **Working dilution** |
| --- | --- | --- | --- | --- |
| **1.** | FOXM1 | Cell Signaling Technology, USA; #5436 | D12D5; Rabbit mAb | 1:1,000 |
| **2.** | Cleaved Caspase-3 (Asp175) | Cell Signaling Technology, USA; #9664 | 5A1E; Rabbit mAb | 1:1,000 |
| **3.** | BCL2A1 | Cell Signaling Technology, USA; #14093 | D1A1C, Rabbit mAb | 1:1,000 |
| **4.** | MCL1 | Cell Signaling Technology, USA; #39224 | D5V5L, Rabbit mAb | 1:1,000 |
| **5.** | BCL2 | Santa Cruz Biotechnology, USA; # SC-7382 | C-2, Mouse mAb | 1:1,000 |
| **6.** | β-actin | Thermo Fisher, USA; # MA1-91399 | AC-15, Mouse mAb | 1:10,000 |
| **7.** | Anti-Rabbit IgG, HRP-conjugated | Jackson Immunoresearch, USA;  #611-035-215 | 611-035-215; Alpaca polyclonal | 1:10,000 |
| **8.** | Anti-Mouse IgG, HRP-conjugated | Jackson Immunoresearch, USA;  #715-035-150 | 715-035-150; Donkey polyclonal | 1:10,000 |
